# The Recruitment-Secretory Block (“R-SB”) Phenomenon and Endoplasmic Reticulum Storage Diseases

**DOI:** 10.3390/ijms22136807

**Published:** 2021-06-24

**Authors:** Francesco Callea, Paolo Tomà, Emanuele Bellacchio

**Affiliations:** 1 Department of Histopathology, Bugando Medical Centre, Catholic University of Healthy and Allied Sciences, Mwanza P.O. Box 1464, Tanzania; 2 Dipartimento Diagnostica Immagini, Bambino Gesù Childrens’ Hospital, IRCCS, Piazza S. Onofrio 4, 00165 Roma, Italy; paolo.toma@opbg.net; 3 Area di Ricerca Genetica e Malattie Rare Bambino Gesù Children’s Hospital, IRCCS, Piazza S. Onofrio 4, 00165 Roma, Italy

**Keywords:** secretory proteins, acute phase reactants, alpha-1-antitrypsin deficiency, hereditary hypofibinogenemia, hepatic storage

## Abstract

In this article, we review the biological and clinical implication of the Recruitment-Secretory Block (“R-SB”) phenomenon. The phenomenon refers to the reaction of the liver with regard to protein secretion in conditions of clinical stimulation. Our basic knowledge of the process is due to the experimental work in animal models. Under basal conditions, the protein synthesis is mainly carried out by periportal (zone 1) hepatocytes that are considered the “professional” synthesizing protein cells. Under stimulation, midlobular and centrolobular (zones 2 and 3) hepatocytes, are progressively recruited according to lobular gradients and contribute to the increase of synthesis and secretion. The block of secretion, operated by exogenous agents, causes intracellular retention of all secretory proteins. The Pi MZ phenotype of Alpha-1-antitrypsin deficiency (AATD) has turned out to be the key for in vivo studies of the reaction of the liver, as synthesis and block of secretion are concomitant. Indeed, the M fraction of AAT is stimulated for synthesis and regularly exported while the Z fraction is mostly retained within the cell. For that reason, the phenomenon has been designated “Recruitment-Secretory Block” (“R-SB”). The “R-SB” phenomenon explains why: (a) the MZ individuals can correct the serum deficiency; (b) the resulting immonohistochemical and electron microscopic (EM) patterns are very peculiar and specific for the diagnosis of the Z mutation in tissue sections in the absence of genotyping; (c) the term carrier is no longer applicable for the heterozygous condition as all Pi MZ individuals undergo storage and the storage predisposes to liver damage. The storage represents the true elementary lesion and consequently reflects the phenotype-genotype correlation; (d) the site and function of the extrahepatic AAT and the relationship between intra and extracellular AAT; (e) last but not least, the concept of Endoplasmic Reticulum Storage Disease (ERSD) and of a new disease, hereditary hypofibrinogenemia with hepatic storage (HHHS). In the light of the emerging phenomenon, described in vitro, namely that M and Z AAT can form heteropolymers within hepatocytes as well as in circulation, we have reviewed the whole clinical and experimental material collected during forty years, in order to evaluate to what extent the polymerization phenomenon occurs in vivo. The paper summarizes similarities and differences between AAT and Fibrinogen as well as between the related diseases, AATD and HHHS. Indeed, fibrinogen gamma chain mutations undergo an aggregation process within the RER of hepatocytes similar to AATD. In addition, this work has clarified the intriguing phenomenon underlying a new syndrome, hereditary hypofibrinogenemia and hypo-APO-B-lipoproteinemia with hepatic storage of fibrinogen and APO-B lipoproteins. It is hoped that these studies could contribute to future research and select strategies aimed to simultaneously correct the hepatocytic storage, thus preventing the liver damage and the plasma deficiency of the two proteins.

## 1. Introduction

The liver synthesizes the vast majority of secretory plasma proteins and represents the only source of circulating alpha-1-antitrypsin (AAT) and fibrinogen [1]. Hepatocytes are regarded as constitutive secretory cells. They synthesize continuously and then secrete plasma proteins into the circulation.

In contrast to regulated secretory cells, mostly hormone-secreting cells, that store the secretion products intracellularly until appropriate extracellular or metabolic signals cause the release of the vesicle content to the exterior, hepatocytes apparently lack the machinery for intracellular storage of proteins destined for secretion, and proteins are secreted almost as fast as they are synthesized [1,2].

The pathway of secretion is common to all secretory proteins either acute or non-acute phase reactants [3,4,5]. Individual proteins are secreted at one of three characteristic rate, classified as fast, intermediate and slow and correspond to the specific intracellular retention half-times of these proteins. The rapidly secreted proteins include albumin and alpha-1-protease inhibitor whilst the slowest include fibrinogen [6]. Experimental animal models have shown that: (i) hepatocytes exert the protein synthesis to different degrees, according to the lobular gradients and intensity of stimuli [7]; (ii) they synthesize more than one acute phase protein simultaneously [8] (iii) the block of secretion by exogenous agents results in a collective retention of all secretory proteins. This information has contributed to understand the mechanisms for acquired protein retention in humans as well but, unfortunately, the rationale is inadequate to explore the retention mechanism of mutant proteins occurring in Hepatic Endoplasmic Reticulum Storage Diseases (ERSD), i.e., Alpha-1-antitrypsin Deficiency (AATD) and Hereditary Hypofibrinogenemia with Hepatic Storage (HHHS) [9].

To that purpose, we found that partial AATD corresponding to the heterozygous Pi MZ phenotype, under conditions of clinical stimulation, fulfills all requirements to study the increase in synthesis and the block of secretion simultaneously. Indeed, in Pi MZ individuals, the secretory block is intrinsic in the Z mutation and does not require external intervention.

## 2. The Secretory Pathway of the Normal and Mutant AAT

The biosynthesis of glycoproteins has been clarified by the work of George Palade granted by the Nobel Prize [10], further complemented by David Sabatini [11] and Gunther Blobel [12]. To the purpose of this review it could be sufficient to refer to the paragraph on biosynthesis of glycoproteins in general, present in another article of this issue of IJMS [13].

Secretory proteins are synthesized in the rough endoplasmic reticulum (RER) and discharged into its lumen. The first sugar attachment (“core glycosylation”) takes place during elongation of the nascent polypeptide chain. Further glycosylation is brought about by glycosyltransferases located in the smooth endoplasmic reticulum (SER) cisternae where trimming and elongation occur. Terminal glycosylation takes place in the Golgi apparatus. Once the last oligosaccharide (sialic acid) is apposed, the mature glycoproteins are available for export through secretory vesicles (SV), driven by microtubules.

Alpha-1-antitrypsin (AAT), the major serine proteinase inhibitor (Pi) in serum, is an acute phase reactant. AAT is a highly polymorphic, more than 100 variants being detected on Isoelectric Focusing (IF). The variants are designated by alphabet letters: the normal variant was called M, whilst the first deficient variant was called Z, due to the slowest isoelectric focalization speed. For the particular mutation, the Z molecule is abnormally conformed and is retained within the cell instead of being regularly exported. Two codominant alleles encode each fraction of the protein. Therefore, normal MM phenotype individuals synthesize and regularly export two pools of M protein (50% each allele).

**Figure 1:**
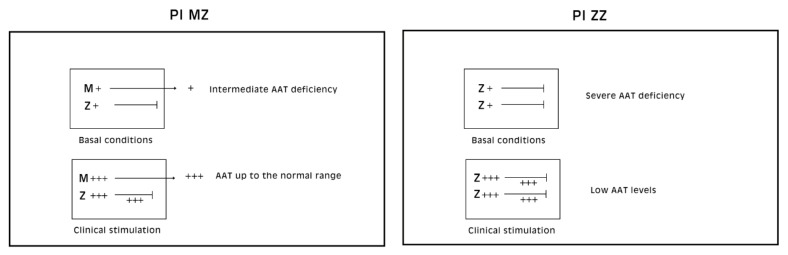
Schematic representation of “R-SB” phenomenon in Pi MZ and Pi ZZ phenotypes. Pi MZ individuals under basal conditions have partial (intermediate) AATD, as the M fraction is regularly exported whilst the Z fraction is retained within hepatocytes. Under conditions of clinical stimulation, the M fraction is increased in synthesis and export and raises the serum AAT levels up to the normal range. In the same time, the intracellular storage of the Z fraction increases; PiZZ individuals have low serum AAT levels either under basal or stimulatory conditions.

Pi MZ livers in basal conditions show intermediate serum values as the M fraction is regularly exported, while 85% of the Z is retained (Figure 1a). Under conditions of clinical stimulation such as inflammation or hormonal stimuli, the synthesis of both M and Z increases. The M fraction is regularly secreted, whilst the Z fraction is retained within the cell. The divergent destiny of the two fractions explains the paradoxical result that the more the serum increases the more hepatocytic storage is (Figure 1a). In contrast, homozygous Pi ZZ individuals show low serum levels in both basal and stimulatory conditions (Figure 1b).

### 2.1. Acquired Defects of Hepatic Protein Secretion

Acquired defects of protein secretion are due to dysfunction of the cell machinery apparatus. They may occur at any step of the pathway, mostly due to exogenous hepatotoxins, alcohol being the prototype. The mechanism of defective secretion in liver cells has been studied in the rat colchicine experimental animal model. Colchicine depolymerizes tubulin and destabilizes the structure and function of microtubules. The loss of function prevents the migration of secretory vesicles (SV) to the plasma membrane thus blocking the last step of secretion. In this experimental model, the retention is characterized by three main features: (a) several proteins are involved simultaneously (so called collective retention); (b) the effect is transitory; (c) the process is reversible and does not have a pathogenetic meaning.

In humans, a similar effect may occur with acute alcohol intoxication. Acetaldehyde, an alcohol metabolite, causes microtubule damage and hampers the migration of SV towards the cell membrane. The secretory proteins are consequently retained collectively along the secretory pathway, stagnate within the ER cisternae and cause dilatation and intracellular inclusions, visualized in routine histological sections [14].

### 2.2. Experimental Models for Acute Phase Proteins in Animals and Human

**Figure 2:**
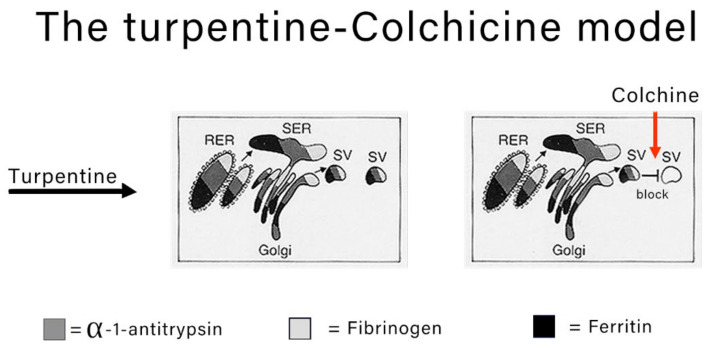
Schematic representation of stimulation and block of secretion in animal models. Turpentine stimulates the liver to increase the synthesis of acute phase reactants (AAT, fibrinogen, ferritin). The proteins translocate from the RER to the SER, Golgi apparatus and SV. The injection of colchicine blocks the migration of SV towards the plasma membrane hampering the export. That results in a collective retention of the proteins within the entire pathway.

The classical experimental model to study the morphodynamics of synthesis and block the secretion of acute phase proteins is the turpentine-colchicine model. Subcutaneous turpentine injection induces an inflammatory reaction followed by a rapid increase in the synthesis of acute reactants and their serum concentration elevation [7,8,15]. The subsequent intraperitoneal injection of colchicine blocks the export of the proteins as shown in Figure 2.

Pi Z transgenic mice have been used to reproduce the AATD human disease (17) but experiments of stimulation and block of secretion have not been performed in that model.

Obviously, the turpentine-colchicine model is not suitable to study the intrahepatocytic fate of the Z AAT. Looking for another model, we came across the human AATD partial deficiency (Pi MZ phenotype) and we found that it is a powerful model to study in onetime stimulation of synthesis and block of secretion, because, due to the co-existence of two codominant encoding alleles, each hepatocyte synthesizes both the normal M and the abnormal Z AAT. The Pi MZ model has turned out to be a sort of Experiment of Nature and has led to rationalize a new phenomenon in biology.

### 2.3. The Hepatocytic Storage in AATD

The heterozygous condition of AATD is of particular interest from both a clinical and biological point of view. Indeed, individuals with this phenotype have partial serum AAT deficiency under basal conditions but are normal or higher than normal under acute phase reaction. For that reason, they are not clinically detectable on the basis of the sole quantitative serum determination and can be diagnosed only on liver tissue specimen examination because of the storage process.

To explain the behavior of AAT in Pi MZ individuals, we have reviewed a pilot study performed on Pi MZ patients and two control groups [16,17,18,19]. Pi MZ patients comprised of 14 individuals affected by malignancy or systemic diseases (Group 1), as a part of a large series of lymphoma patients [20], and 13 patients affected by chronic liver disease (Group 2), a condition previously shown to be able to raise the serum AAT level [21]. The control groups consisted of 30 Pi MM patients affected by malignant lymphoma (Group 3) and 8 Pi ZZ patients (Group 4) with chronic liver disease. From all patients, one or serial liver biopsies, the Pi phenotype and serum AAT values at the time of the liver biopsy were available.

All patients from Groups 1 and 3 showed higher than normal serum levels, 5 out of 13 from Group 2, and 8 out 8 from Group 4 had lower than normal serum AAT levels (Table 1).

Tissue sections from all biopsies were stained by immunohistochemistry with both polyclonal [16,17,18] and the AZT11 monoclonal [19], specific for the Z protein.

**Figure 3:**
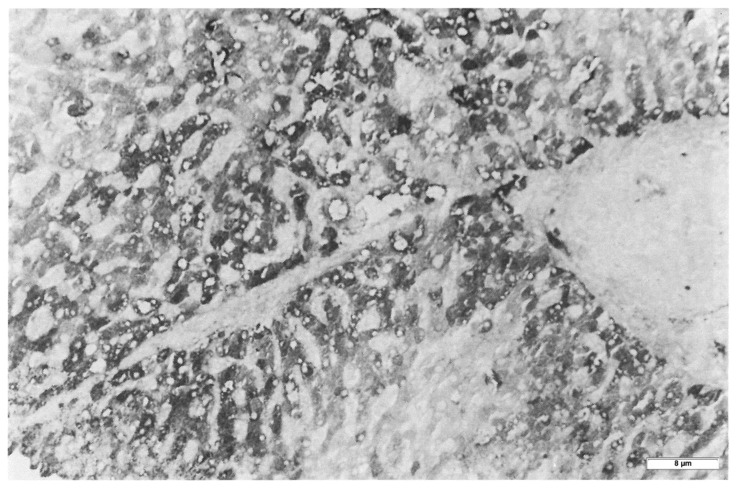
Freshly frozen liver tissue specimen from a Pi MM lymphoma patient, stained with a polyclonal anti-AAT antibody. The positivity involves the periportal hepatocytes and extends to the whole zone 1 of the liver acini. The positivity appears as diffuse cytoplasmic indicating the presence of the protein within the entire secretory pathway. Portal tracts are enlarged, rounded and infiltrated by lymphomatous cells. (AAT immunoperoxidase staining on frozen section × 320) (Figure from Callea F. PhD Thesis, Acco, Leuven, 1983: 1–153).

Hepatocytes from MM (Group 3) biopsies were always negative with either the polyclonal or the monoclonal antibody. The M AAT cannot be visualized by immunohistochemistry in tissue sections due to the rapid synthesis and export of the protein that leaves the intracellular concentration below the threshold of sensitivity of the technique pertaining the M protein. The latter can be detected only in sections from freshly frozen tissue (Figure 3) [17]. Likewise, the M AAT could not be visualized under the EM owing to its transparency and solubility in the ER milieu [15].

**Figure 4:**
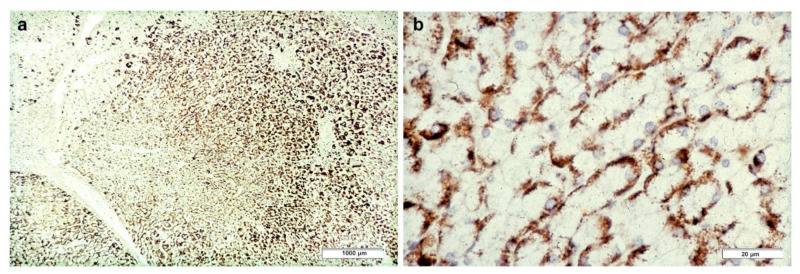
Pi MZ liver specimen. All hepatocytes from adjacent lobules are positively stained for AAT (**a**) polyclonal AAT immunostaining × 2. The intensity of the staining decreases from zone 1, where the positivity involves the whole cytoplasm (type I positivity), to zones 2 and 3 where crescent like and rectilinear double rows pictures are clearly seen. (**b**) polyclonal AAT immunostaining × 100.

In Groups 1 and 2, all hepatocytes (up to 100%) could be positive (Figure 4a). The positivity of periportal hepatocytes are depicted globules or granules distributed in the whole cytoplasm (type I positivity), whilst in midlobular and centrolobular hepatocytes, the positivity appeared in the form of crescent-like or rectilinear double row arrays beneath the plasma membrane along the sinusoidal wall (type II positivity) (Figure 4b). The intensity of the staining was decreasing from zone 1 to zone 3 of the lobules. The AAT inclusions were negative for other secretory proteins, indicating that only the Z protein was retained [19].

**Figure 5:**
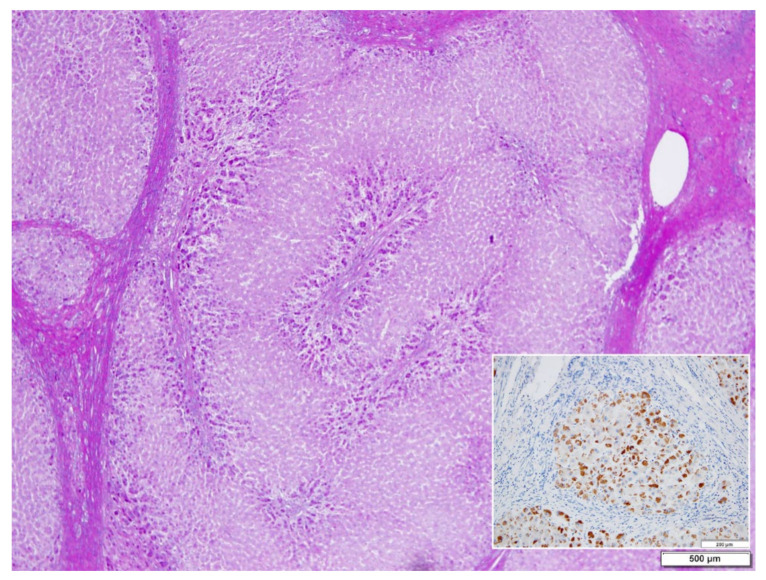
Pi ZZ explanted liver. A mixed type (uni- and multilobular) cirrhosis. PASD inclusions are present in zone 1 hepatocytes, separated from the connective tissue of the portal tracts by edema. PAD × 1.25. AAT inclusions fill up the entire hepatocytic cytoplasm in a small nodule. A few positive cells are scattered in the central part (Inset. AAT polyclonal immunostaining × 60).

In ZZ patients from groups 2 and 4, the positivity was mainly restricted to zone 1 (periportal) hepatocyte in the form of PAD positive globules (Figure 5) filling up the entire cytoplasm (type I positivity), or unevenly distributed in the intralobular hepatocytes with type II positivity (Figure 5 inset).

**Figure 6:**
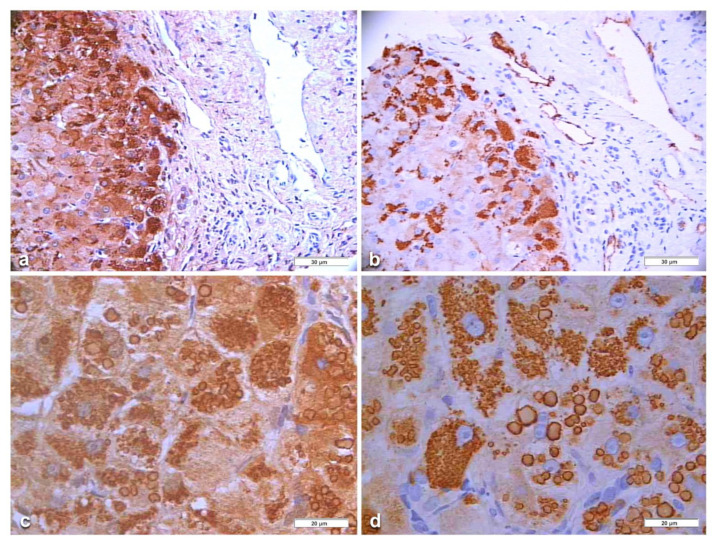
Pi MZ liver. AAT immunoreactive inclusions fill up the entire cytoplasm of periportal hepatocytes (**a**) polyclonal anti-AAT immunostaining 40×. A consecutive section stained with a monoclonal AZT antibody shows less intense staining. Single inclusions have a sharply demarcated periphery. Endothelial cells of portal vessels are stained by the monoclonal but not by the polyclonal one (**b**) monoclonal AZT antibody 40×. Two consecutive sections from a Pi MZ liver. Both sections show a high degree of storage and large size of globules. The staining intensity of the polyclonal-AAT section appears higher with the AAT polyclonal (**c**) immunostaining × 100 than with the monoclonal (**d**) immunostaining 100×.

In Pi MZ patients, the polyclonal antibody gave a stronger positivity than the monoclonal and apparently involved more hepatocytes (Figure 6a–d). The monoclonal AZT 11 antibody stained not only hepatocytes but also endothelial cells of portal vessels (Figure 6b). We could not perform a double immunostaining for simultaneous visualization of the two AAT fractions because of the unavailability of a monoclonal anti-M AAT.

**Figure 7:**
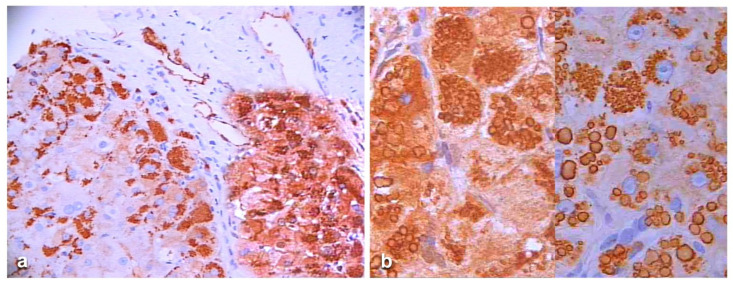
The polyclonal stained section (box) has overridden the portal tract of the monoclonal one where endothelial cells are positive to the AZT 11 antibody. (**a**) Photo collage of two consecutive immunostained sections subtracting the monoclonal from the polyclonal stain (**b**).

In an attempt to objectivize the different staining intensities between the two antibodies, we could only have a recourse to a graphic stratagem by working out with Figure 6, by overriding the polyclonal stained section (box) over the portal tract of the monoclonal one where endothelial cells are positive to AZT11 antibody Figure 7a), and by making a photo collage subtracting the monoclonal from the polyclonal stain (Figure 7b)

**Figure 8:**
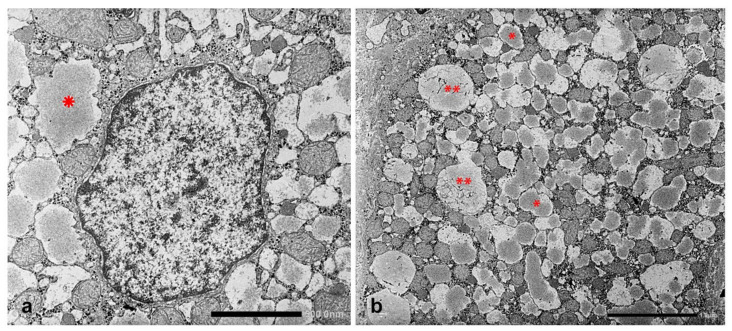
PiMZ phenotype patient. The electronmicrophotograph shows a periportal hepatocyte with dilated cisternae of the RER containing amorphous fluffy AAT material (*). RER membranes are in close contact with mitochondria (**a**) (EM × 15.725). The electronmicrophotograph shows a large cytoplasmic portion of a hepatocyte with diffuse dilatation of ER cisternae. Most of the AAT-like material appears in the form of amorphous semi-electron dense material (*). A few inclusions present a dense, compact appearance and entrapped remnants of disrupted membranes (**) (**b**) (EM × 7820).

In Groups 1, 2 and 4 biopsies, under the EM, AAT appeared in the form of fluffy amorphous material within the lumen of dilated cisternae of the RER (Figure 8a). In periportal hepatocytes, AAT could also present a more compact, dense appearance (Figure 8b).

**Figure 9:**
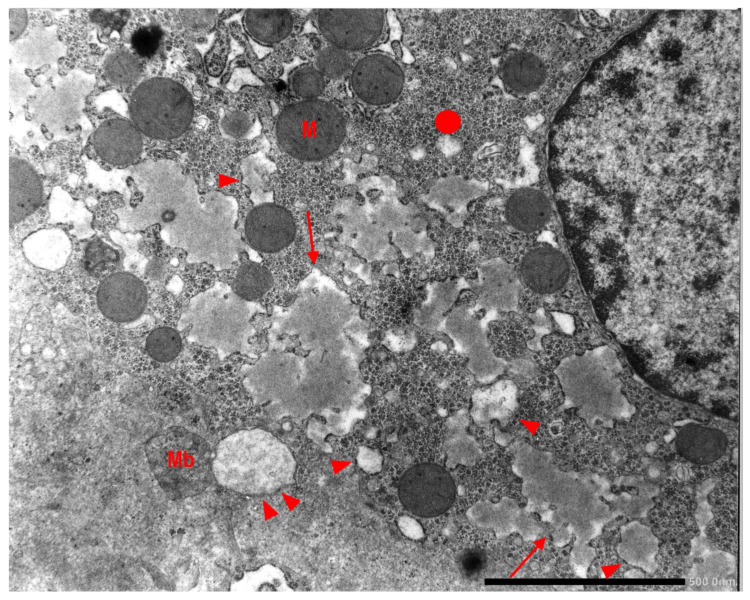
Pi MZ patient liver. The electronmicriphotpgraph shows AAT like material in dilated cisternae of the RER. The intraluminal material appears in the form of addensed granules. In a few cisternae the material is loose and made up of fragmented tangle filaments. This feature is more obvious in dilated cisternae nearly empty (single arrowheads and double arrow-head). The hepatocyte contains abundant glycogen (circle), mitochondria (M) and a multivesiculr body (Mb) (EM × 15.725).

In Pi MZ livers the intralumenal AAT could present a yet undescribed feature made up of fibrils, and tangles of filaments (Figure 9).

Similar features had been described in an experimental work at very high EM magnification in AAT inclusion bodies extracted from a Pi ZZ liver [22] and more recently confirmed also in a Pi MZ explanted liver (24).

**Figure 10:**
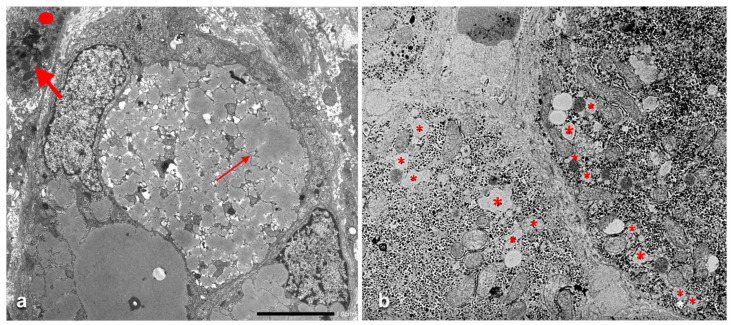
Pi MZ patient. The electronmicrophotograph shows dilated channels of the RER filling up the entire cytoplasm (**a**) EM × 14.500. Dilated channels coalesce in larger cisternae (arrow). This feature corresponds to type I immunohistochemical positivity. The electronmicrophotograph shows mildly dilated cisternae of the RER (*), located at the periphery of the cytoplasm of two adjacent hepatocytes, representative of type II positivity in immunostaining (**b**) EM × 11.730.

The AAT containing cisternae were distributed in the entire cytoplasm in type I positive hepatocytes (Figure 10a), while in hepatocytes with immunohistochemical type II positivity, the RER with intralumenal AAT was found only at the periphery of the cytoplasm (Figure 10b).

### 2.4. The Recruitment-Secretory Block (“R-SB”) Phenomenon

The “R-SB” phenomenon has been described in 1983 in a PhD thesis [17] and published in an International Journal in 1984 [16].

**Figure 11:**
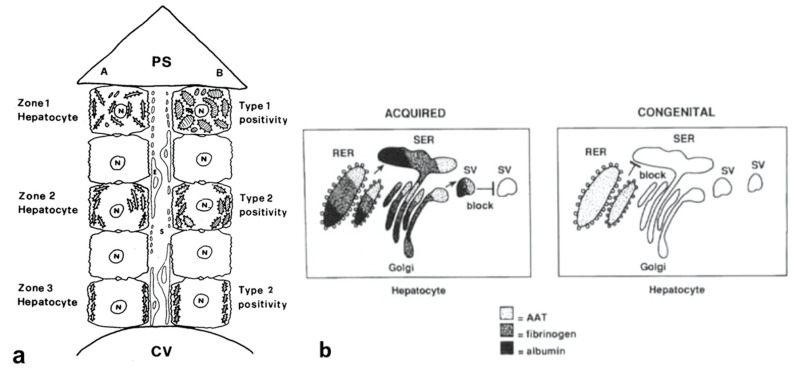
Schematic representation of amount and distribution of the RER in hepatocytes, and “R-SB” phenomenon in hepatic lobule, according to a lobular (portal-central) gradient. A and B represent liver cell muralia. PT = portal tract. CV = central vein. The full cytoplasmic granular staining corresponds to type I positivity. Crescents and double row staining correspond to type II positivity. (**a**) Schematic representation of acquired vs. congenital defects of protein secretion. The acquired defects of protein secretion are due to exogenous agents that block the migration of secretory vesicles (CV) and cause a collective retention of secretory proteins. The congenital defects affect mutant proteins and cause a selective and exclusive retention of a given mutant protein in the early stage of synthesis within the RER (**b**).

The identification of the phenomenon has been made possible by comparing the serum and morphological findings in Pi MZ phenotype with the amount and anatomical distribution of the RER in the normal human liver [23], as schematically represented in Figure 11a. The RER indeed is abundant in zone 1 hepatocytes and fills up the entire cytoplasm. That explains why the retained AAT results in type I positivity in immunostained preparations. The RER is less represented in midlobular and centrolobular hepatocytes where it is marginalized towards the cell periphery beneath the plasma membrane. Thus the retained AAT in these cells appear in the form of crescents or rectilinear double rows (type II positivity).

As type II positivity occurs in all Pi MZ cases with normal or elevated serum AAT levels, it identifies the newly recruited cells for synthesis during acute phase stimuli, whilst type I positivity is commonly observed in periportal hepatocytes that are predisposed to chronic engulfment as they synthesize the protein continuously. For that reason, immunostaining and EM patterns have been considered as expression of simultaneous recruitment of new hepatocytes for synthesis of both M and Z AAT and of secretory block of the Z protein. The phenomenon has been labelled as “Recruitment-Secretory Block” (“R-SB”) and type II positivity proposed as its hallmark [16].

In other words, the R-SB phenomenon turns out to reveal the extent of engagement in the synthesis and the extent of derangement of the liver in the secretion of AAT.

The AAT immunostaining pattern in PI MZ individuals reflects not only the amount and the distribution of the RER in the normal human liver according to Ma and Biempica (Figure 11a), but also the concepts of heterogeneity of parenchymal liver cells [24] and of streaming liver [25]. The abundance of RER in periportal hepatocytes reflects their commitment to a major protein synthesis as “routine” or “professional” protein synthesizing cells, whilst midlobular and centrolobular hepatocytes have less RER and more SER because they are involved in other metabolic functions, especially bile synthesis and microsomal enzyme activity [26,27].

The “R-SB” phenomenon has definitely clarified the difference between acquired and congenital defects of protein secretion. In the former, exemplified by the turpentine/colchicine model, the block is due to an exogenous agent and causes a collective retention of secretory proteins, whilst in Pi MZ individuals, the secretory block is intrinsic in the mutant Z protein and results in a selective retention (Figure 11b).

### 2.5. The “R-SB” Phenomenon and M and Z Heteropolymers

The described “R-SB” phenomenon is in agreement with the concept that the plasma levels in Pi MZ heterozygotes result from independent secretion of the two variants (24).

However, in Pi MZ individuals, the immunostaining of liver sections with a polyclonal anti-AAT antibody, which recognizes all AAT variants, has shown a higher amount of retained protein as compared to the monoclonal anti-Z AAT antibody, which recognizes exclusively the Z fraction.

This observation per se would suggest that the immunoreactive inclusions could contain not only Z but also M AAT.

Interestingly enough, the positive reaction obtained only in freshly frozen Pi MM livers, appeared as a full cytoplasmic staining, indicating that the protein was located within the entire secretory pathway (Figure 3). This pattern is quite analogous to that obtained in experimental animal models for protein secretion [17].

The authenticity of the immunomorphological findings in Pi MZ livers, has been confirmed by comparing consecutive sections alternatively stained. Whether these findings allow the interpretation of a colocalization of Z and M AAT in the form of heteropolymers is highly suggestive in view of recognized phenomenon of AAT heteropolymerizations [28], recently culminated in the demonstration that Z AAT can form heteropolymers with wild type M AAT, thus leading to an increased intracellular concentration of M AAT. That is the requirement for detecting M AAT in paraffin embedded material with a polyclonal anti-AAT antibody.

In that respect, it is worth mentioning the positive immunoreaction we observed in endothelial cells of portal tract vessels with the monoclonal ATZ 11 antibody that, according to Janciauskiene et al., detects circulating and endothelial cell polymers of Z and wild-type AAT [29]. Secretion of Z AAT polymers in cell models [30] and circulating polymers in AATD deficiency has been also demonstrated [31].

The ability to form heteropolymers within cells most likely reflects the capacity of each protein to form an unstable intermediate conformer (M*) which acts as a nucleus for heteropolymer formation [22,28]. As no interaction occurs when the two proteins are incubated together in vitro (31), it is suggested that heteropolymer formation must occur while the M and Z are folding within the cell rather than from native conformers of protein. The co-localization of M and Z AAT in the aggregates within the ER cisternae has been shown in spectacular confocal microscopy images [28] by using, in combination, the following monoclonal antibodies: (a) anti-total AAT mAb3CH, anti-polymeric mAb 2c1 [32]; anti mAb2H2 specific for the M variant.

We believe that our immunohistochemical findings represent the in vivo macroscopic counterpart of the heteropolimerization of AAT mutants observed in cell models mimicking heterozygosity. In other words, from the immunohistochemical results, it would seem that the polyclonal AAT antibody in Pi MZ individuals is staining the co-localized M and Z component.

**Figure 12:**
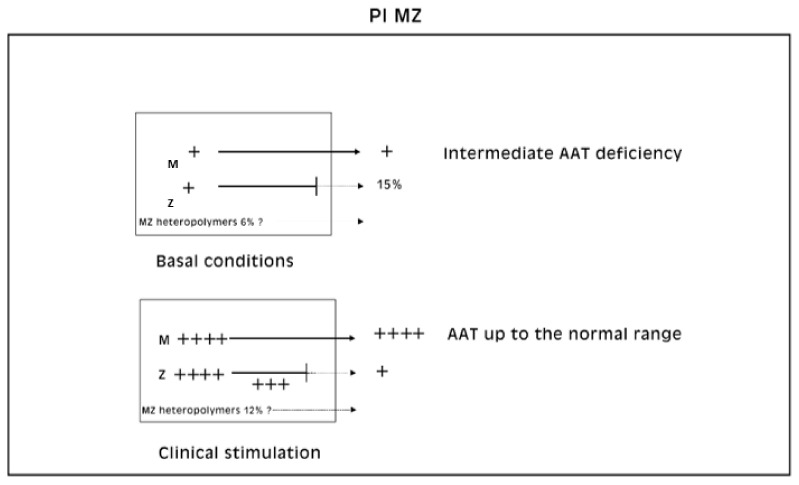
Schematic representation of the “R-SB” phenomenon in Pi MZ phenotype in the light of M-Z heteropolymerization. By comparing this scheme with that in Figure 1a, it appears the in basal condition, the total amount of AAT results from M, Z and M-Z polymers, respectively estimated as 50%, 15%, 6% (upper panel). Under conditions of clinical stimulation, presumably, there is an increase of the percentage of all components (lower panel).

Laffranchi et al. were able to calculate that M AAT comprises around 6% of the polymer subunits in the MZ liver sample [28]. Our methodology is not adequate to predict to what extent the M AAT can co-polymerize or whether the rescue phenomenon works for either proteins. A rough estimation of the co-polymerized M AAT could be only obtained by detraction from the two immunostainings (Figure 12).

Taken all together, the available data allow to forward the following hypothesis on M-Z aggregation and Z (vs M) rescue, expressed into two cartoons.

**Figure 13:**
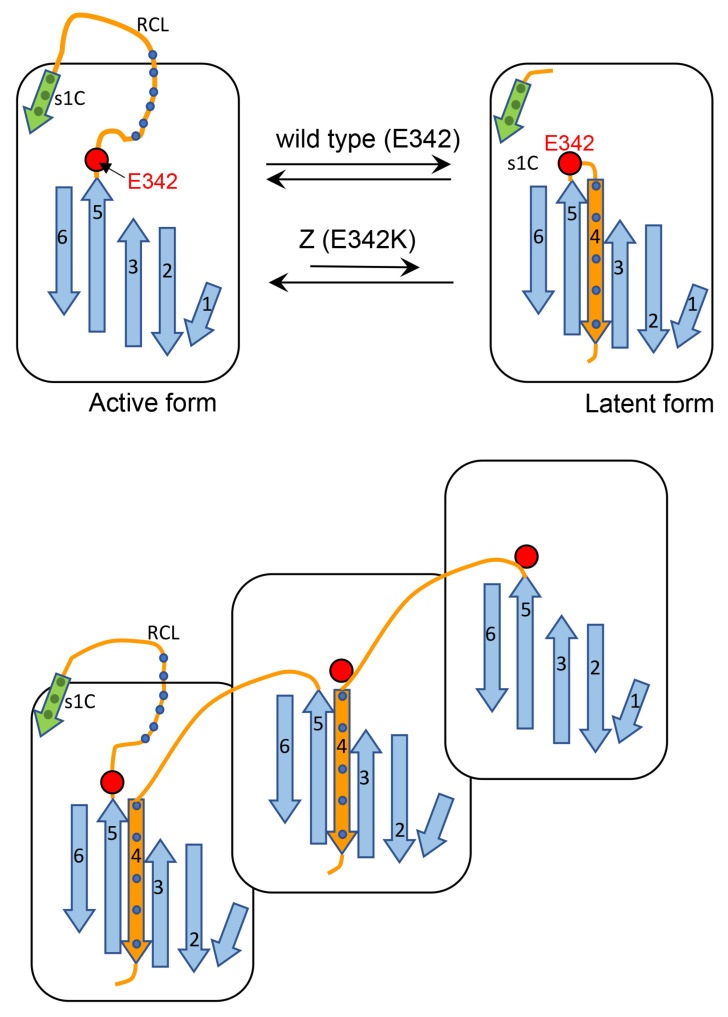
Schematic representation of the intramolecular insertion of the polymerized RCL peptide into the beta-sheet of AAT between beta—strand 5 with the rest of the protein structure. This results in a decreased function as a hinge of the affected site makes the thermodynamic equilibrium of the intramolecular RCL insertion less favorable (**Upper panel**). The alternative intermolecular RCL insertion allows the mutant to produce elongating multi-AAT structures. Also M AAT can engage in intermolecular insertion forming mixed M-Z aggregates, but it can dynamically escape by switching to more stable intramolecular RCL insertion, which reverses the growth of aggregates and partially rescues Z AAT (**Lower panel**).

Cartoon A (Figure 13) shows that after proteolytic cleavage, the reactive center loop (RCL) is inserted as a new β -strand (β-strand 4) into the middle of the β-sheet A. This process is affected in the Z AAT since the mutations are known to cause lability in the interaction between the extremity of the affected β-strand 5 and the rest of the protein. Thus, we propose that this mutation by loosening the hinge region of RCL, decreases its ability to find the correct path for intramolecular insertion in the form of β-strand 4.Cartoon B (Figure 13) shows an alternative process whereby the RCL undergoes a similar mechanism of insertion in the middle of β-sheet A but on a different AAT molecule and this might explain the higher tendency of Z to aggregate. M can also be engaged in this process, however, since this AAT molecule is more efficient in inserting, intramolecularly, the RCL into the β-sheet, it competes with the abnormal intermolecular insertions and co-polymer elongation due to the Z molecules. M molecules that have already been recruited into aggregates with Z AAT might be able to switch from the intermolecular to the intramolecular insertion of β-strand 4 thus breaking the heteropolymers. Therefore, the co-participation of M in aggregations with Z decreases the stability of the mixed M-Z assemblies and can also partially rescue Z, improving its secretion.

Studies on AAT polymer formation have been performed on polymers extracted from the liver tissue of a MZ AAT heterozygote by using a conformationally developed antibody with selectivity for M AAT with respect to Z AAT. The rationale of that in vitro model is comparable to the in vivo “R-SB” phenomenon in Pi MZ phenotype. Moreover, the results appear to be in congruence. The final proof about the equivalence would come from prospective studies on Pi MZ livers, by using anti-Z and anti-M monoclonal antibodies in consecutive sections or in double immunostaining on the same section.

## 3. The Byosynthesis of Fibrinogen

The liver is the only source of fibrinogen in humans. Here we review, in a telegraphic way, the biosynthesis of fibrinogen in order to understand the mechanism of aggregation in case of mutations.

The circulating fibrinogen is a hexamer made up of two sets of symmetrical trimers, each formed by A-alpha, B-beta and gamma chains respectively encoded by FGA, FGB and FGG genes. The hexamer molecule contains a central E region and two lateral regions, corresponding to the globular D domain. The D domain is crucial for D:D formation. Indeed, all eight mutations so far described in the globular domain of the gamma chain [13,33,34] induce important changes that hamper the gamma dimer formation and cause catastrophic effects consisting conformational abnormalities of the molecule that trigger intracellular aggregation with a modality explained by 3-D studies [34,35]. The single fibrinogen mutation occurring in the A-alpha chain has not yet been clarified [36].

The assembly of the two trimers takes place in the ER, through a stepwise process, with an initial formation of dimers and a subsequent addition of a third chain followed by the fast integration of the two trimers. Abnormal gamma monomers cause intracellular aggregation and leave exposed hydrophobic patches that become available for undue binding to other hydrophobic molecules, such as APO-B-lipoproteins [37]. After maturation in the Golgi apparatus, fibrinogen is exported into the circulation. The intracellular normal fibrinogen is soluble in the ER milieu. During clot formation, fibrinogen is converted into an insoluble form by thrombin-mediated proteolytic action. Through an intricate stepwise process, single fibrin monomers associate into protofibrils that, in turn, aggregate into fibers thus yielding the stable fibrin meshwork.

### 3.1. The Secretion of Fibrinogen under Acute Phase Stimulation in Experimental Animal Models

The secretion of fibrinogen under acute phase stimuli has been studied extensively in experimental animal models by the turpentine/colchicine model. Subcutaneous injection of turpentine induces an inflammatory reaction. The liver responds by a progressive recruitment of hepatocytes from zone 1 to zone 3 for the synthesis of acute phase proteins. Interestingly, when liver tissue sections are stained in such conditions, hepatocytes show a diffuse cytoplasmic staining as the immunoreaction is depicting the proteins moving along the entire synthetic pathway (RER, SER, Golgi apparatus, SV). This staining pattern is analogous to that observed in frozen sections from Pi MM individuals as shown in Figure 3.

Colchicine, injected intraperitoneally at intervals, depolymerizes hepatocytic microtubules and prevents the migration of SV towards the cell membrane, thus blocking the last step of secretion. That results in the retention of not only fibrinogen but also of all secretory proteins (so called collective retention) (Figure 11b).

In patients affected by HHHS, the mutant fibrinogen is retained within the RER and only a small amount is exported into the circulation. In our original Fibrinogen Brescia mutation, the patient as well as family members, had shown constantly low plasma levels over a 15year follow-up period [38]. In the Trabzon mutation patient, some fluctuations (up to the double) have occurred over the years but plasma levels have always remained three-four-fold below the highest values of the normal range [38].

### 3.2. The “R-SB” Phenomenon in HHHS

HHHS is an extremely rare condition as compared to AATD and occurs only in the heterozygous state. The homozygous condition is believed to be incompatible with life [13]. This background makes the comparison of HHHS with the heterozygous Pi MZ AATD in conditions of clinical stimulation unfeasible.

Moreover, no single HHHS patient has so far developed HCC or systemic diseases that, in heterozygous AATD patients, are associated with serum elevation of AAT due to the “R-SB” phenomenon.

**Figure 14:**
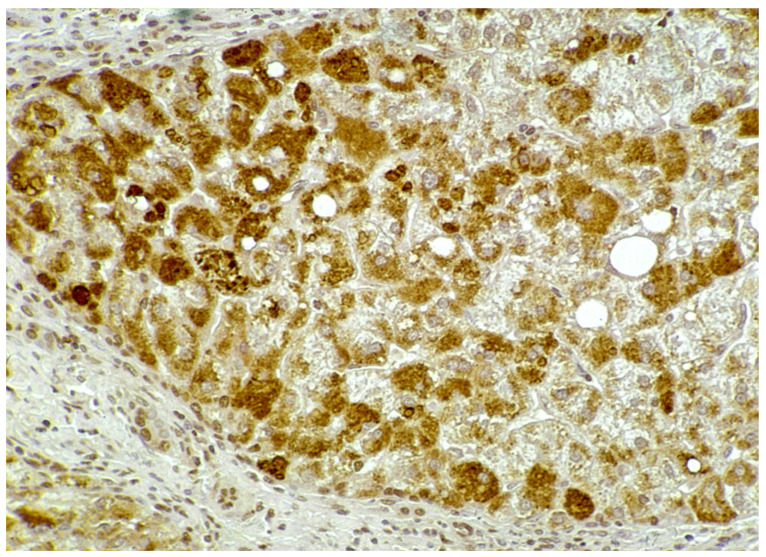
HHHS cirrhotic liver specimen. Hepatocytes in a parenchymal cirrhotic nodule contain inclusions specifically immunoreactive to an anti-gamma chain fibrinogen antibody (monoclonal FGG immunostaining × 100).

Heterozygous HHHS seems to behave like the homozygous Pi ZZ AATD. In both these conditions, the circulating levels of AAT and fibrinogen are very low. Only 10-15% of the Z AAT and less than 10% of fibrinogen are exported but apparently no mutant gamma chains are found in circulation [38,39]. The gamma chain is entirely retained in the polymerized intracellular aggregates together with A-alpha, B-beta, D and E regions as we could demonstrate by using specific monoclonal antibodies against individual fractions (Figure 14) [40].

**Figure 15:**
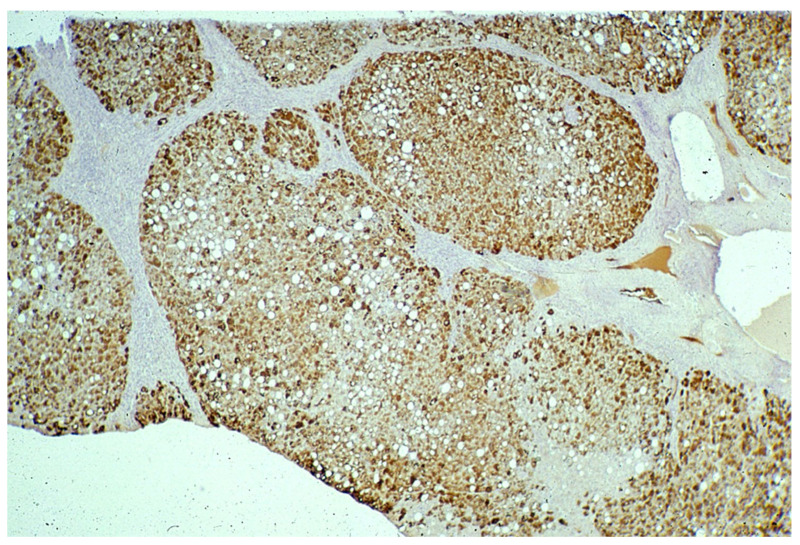
HHHS cirrhotic liver. Fibrinogen inclusions are present in all parenchymal nodules. The immunoreaction appears in the whole cytoplasm. In the central part of the smallest nodule, the positivity appears at the periphery of the cells, beneath the plasma membrane (polyclonal fibrinogen immunostaining × 2).

On immunostaining, HHHS and Pi ZZ patients show a similar pattern especially in the cirrhotic stage. Hepatocytes show mainly type I positivity, but type II can be observed in some areas of the nodules (Figure 15).

Type II positivity depicts midlobular and centrolobular hepatocytes that constitutively contain less amounts of RER localized only at the cell periphery. In analogy to AATD, the retention of fibrinogen in that subcellular location indicates that the RER is the major site for the storage of fibrinogen and that the storage starts in the very early stage of synthesis in freshly recruited hepatocytes for protein synthesis.

**Figure 16:**
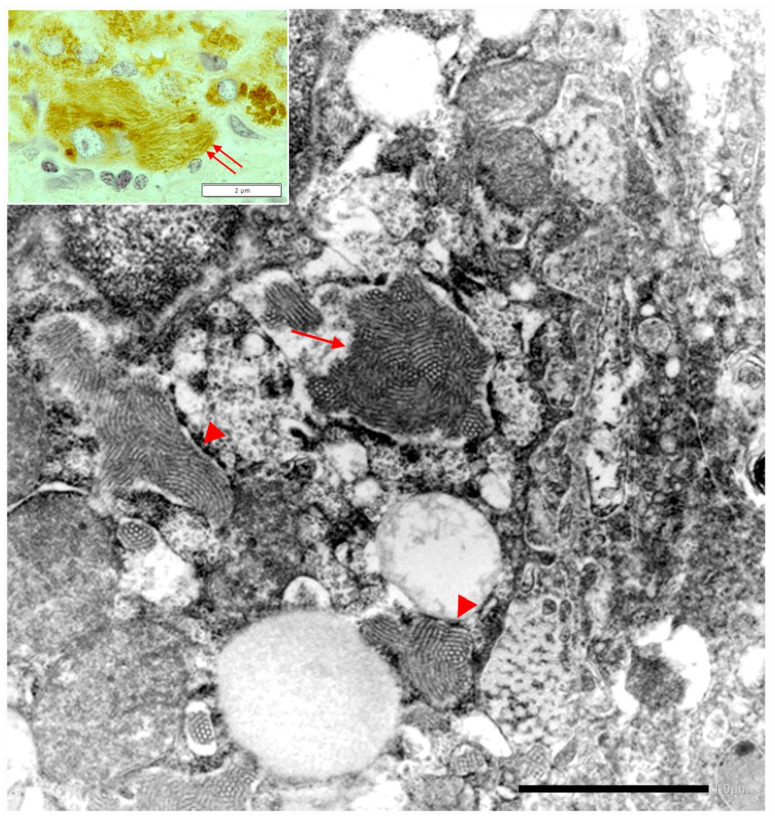
The electronmicrograh shows a hepatocyte with dilated RER containing densely packed tubular structures arranged in curved bundles (finger print-like) (arrow), or elongated fibers with a metameric array (arrow-heads) corresponding to fibrinogen, reminiscent of extracellular fibrin (EM × 8.000). The elongated fibers appear as acicular fiber glass-like inclusions (double arrow) (Inset: FGG immunostaining × 1200).

Under the EM, the mutant fibrinogen in the RER shows a highly specialized organoid appearance resulting from closely packaged curved tubular structures resembling finger print or elongated fibers suggesting fiber glass. The fiber glass inclusions are better visualized in immunostained preparations that highlight the acicular cristal-like configuration (Figure 16).

### 3.3. HHHS and Hypo-Beta-Lipoproteinemia

**Figure 17:**
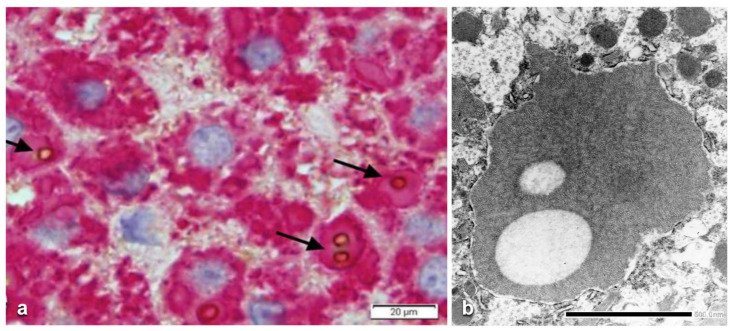
HHHS patient with Hypo-AOOO-B lipoproteinemia. Hepatocytes contain plenty of fibrinogen immunoreactive material (red color), inside fibrinogen inclusions there are rounded inclusions positively stained by an anti-APO-B lipoprotein antibody (arrows). (**a**) double immunostaining × 1000. The electronmicrophotograph shows a single fibrinogen inclusion in a dilated cistern of the RER. The inclusion consists of closely packaged tubular structures with a finger print-like appearance. A lipid droplet is present within the fibrinogen inclusion (**b**) (EM × 31.750).

The association of hypofibrinogenemia and hypo-APO-B-lipoproteinemia has been described in fibrinogen gamma chain mutations [37]. The molecular analysis has revealed no abnormalities in APO-B and MTTP regulatory genes. Interestingly the mutant fibrinogen and the normal APO-B-lipoprotein were co-localized in the same inclusions (Figure 17a) within the hepatocytic RER (Figure 17b) and were deficient in plasma.

All fibrinogen mutations in HHHS are located at the globular gamma domain of the gamma chain involved in the “end-to-end” interaction thus impairing the D-dimer formation. Therefore, each monomeric gamma chain is left with an abnormal exposure of hydrophobic patches that can trigger an irregular recruitment of the hydrophobic molecules APO-B and lipids, causing their intracellular retention and impairment of export [37].

The association between HHHS and hypo-APO-B-lipoproteinemia represents a new syndrome. Interestingly, a physiological link has been demonstrated between the two proteins either in basal or fibrinogen overexpression [41]. APO-B represents the principal constituent of the very low dense lipoprotein (VLDL), and is synthesized in the RER, discharged into the ER lumen, assembled with glycolipids and further secreted as immunoprecipitable VLDL [42]. In experimental animal models for protein secretion block, fibrinogen and lipoproteins does not run in parallel, as the same agent, colchicine, has a double effect on fibrinogen, first inhibiting the synthesis by directly binding to polysomes on the RER, and later on blocking the microtubule function [15], whilst lipoprotein secretion is affected only in the last step of the secretory pathway [43].

The simultaneous retention of abnormal fibrinogen and normal lipoprotein molecules appear to share some analogy with the divergent behavior of M and Z AAT fraction as reflected into the “R-SB” phenomenon, and at the same time with the recent observations showing that about 6% of the normal M AAT can make heteropolymers with the Z AAT in the RER [28] and that these heteropolymers can be found in circulation [28,29,44,45].

Presently no treatments are available for HHHS. Carbamazepine and ursodeoxycholic acid have been used empirically [46,47]. Short term administration has resulted in a decrease in transaminase levels, thus suggesting a potential protective role of those drugs against liver cell damage. However, the impact on plasma levels or amount of fibrinogen storage is missing.

Considering the pathogenesis of the newly described syndrome, one would predict that APO-B-deficiency could be managed by using exogenous small molecules replacing APO-B in binding hydrophobic patches of the abnormal gamma monomers.

In analogy with AATD however a logical rationale would be the exploitation of the formation of intracellular heteropolymers between the two stored proteins and their export in order to obtain serum concentration elevation of both proteins.

Until specific treatment will be available, it could be a challenge to treat HHHS and APO-B by using the same protocol treatment that has been shown to be effective in congenital APO-B deficiency.

## 4. The “R-SB” Phenomenon in Pi Z Transgenic Mice

Pi Z transgenic mice have been used as an animal model for human AATD [48,49]. All studies were able to show storage of the human Z protein in the form of PASD globules corresponding to dilated cisternae of the RER of hepatocytes, but the model is far away from recapitulating the natural history of liver pathology in human AATD [50].

We have reported a morphological and genetic study on a large number of Pi Z transgenic mice [50], and in this paper we have reviewed all the material from that experimental work, with the aim of searching for the morphological counterpart of the “R-SB” phenomenon observed in human AATD.

The rationale of the study was based upon the following statements: (i) Pi Z mice liver synthesizes both normal mouse AAT and Pi Z human AAT; (ii) the normal mouse AAT is regularly exported like the M AAT in humans; (iii) 85% of the Z AAT is retained within the RER of hepatocytes; (iv) there is no immunohistochemical cross-reaction between human and mouse anti-AAT antibodies; (v) both monoclonal and polyclonal antibodies are staining the human Z AAT but not the mouse AAT.

**Figure 18:**
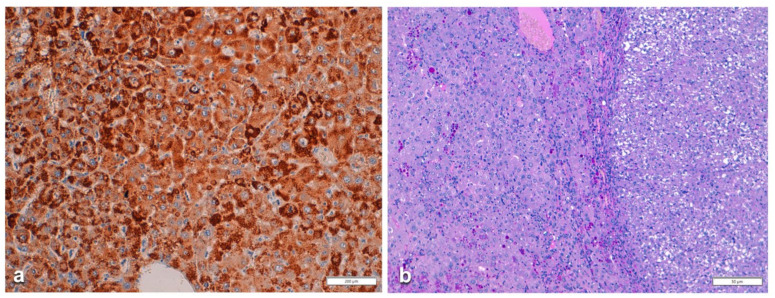
Histological sections from a Pi Z mouse liver. All hepatocytes from the non-tumorous tissue are stained positively for AAT. The positivity is mainly of type I. However, type II positivity is present in the deep part of the lobule (**a**) (Z AAT immunostaining × 100). In Figure b a well demarcated tumor nodule is present. Tumorous hepatocytes are devoid of PAS-D globules, that are observed in the non-tumorous tissue in the left part of the section (**b**) (PASD staining × 6).

In mice with HCC or other malignancies, the AAT staining pattern of the normal parenchyma involved large areas of the lobules either in a confluent or in a streaming-like fashion (pattern A) (Figure 18a). HHC arising in Pi Z mice did not show AAT inclusions similar to HCC arising in AATD patients (Figure 18b) [50,51,52].

**Figure 19:**
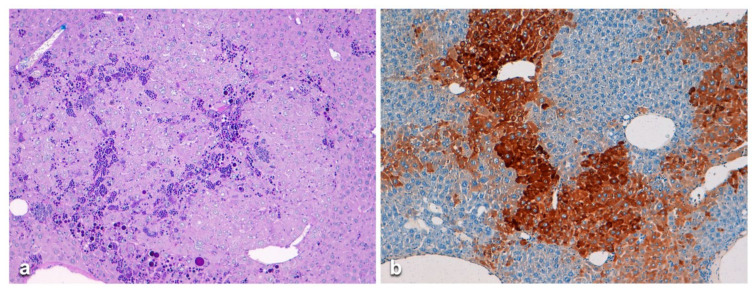
Histological section from a Pi Z mouse liver. The low power view shows preserved lobular architecture in the absence of inflammation or necrosis. Periportal hepatocytes contain PASD globules similar to human Pi Z individuals (**a**) (PASD × 2). The immunostaining for AAT highlights the topographical distribution and the morphology of the globules corresponding to the PASD stain (**b**) (AAT immunostaining × 60).

This intriguing phenomenon has been previously discussed [50]. In mice without tumors, PASD inclusions were patchily distributed and mostly visualized in periportal areas (Figure 19a). The immunostaining has confirmed that the PASD globules contained human Z AAT (Pattern B) (Figure 19b).

Unfortunately, this study has been designed retrospectively, therefore we could not perform serum concentration determination of either protein. However, in the light of the “R-SB” phenomenon, it is reasonable to forward the interpretation of pattern A as an expression of acute phase reaction and of pattern B as an expression of resting or basal conditions.

As the Pi Z mice do not carry the human M allele and polyclonal anti AAT antibody does not cross-react with the mouse AAT, the transgenic mice model is not suitable for polymer or allo- or hetero-polymer formation studies.

The lack of serological data in our study represents a limitation for comparing Pi Z mice and homozygous or heterozygous human AATD. According to Carlson’s data [48], however one can predict that control mice, under acute phase stimuli, are capable of raising their serum AAT level and that Pi Z mice can raise, to some extent, according to our data, that of the human Z AAT.

## 5. Conclusions and Perspectives

In this article, we have shown how experimental animal models have contributed to the understanding of the morphodynamics of the synthesis and block of secretory proteins in liver tissue.

Like in humans, the protein synthesis starts in zone 1 where professionally protein synthesizing hepatocytes are located. Under acute phase stimuli, hepatocytes of zones 2 and 3 are recruited for additional synthesis and rapid export that leads to increased plasma levels. Each hepatocyte is synthesizing the vast majority of acute phase reactants.

The secretory block brought about by colchicine, after turpentine stimulation, results in retention of all proteins. The phenomenon is called collective retention of secretory proteins. The block affects, first the SV and subsequently backwards the Golgi apparatus, SER and RER. The proteins stagnate in the channels of the secretory pathway and give rise to inclusions visualized in light and EM.

The animal model applies to the acquired storage of secretory proteins in human liver after excess alcohol intake or other cytotoxic agents that cause microtubule damage and dysfunction.

However, the model does not apply to the congenital storage of mutant secretory proteins in AATD or HHHS.

To study in the same time stimulation and secretory block of mutant proteins we have selected the Pi MZ phenotype of AATD because of the unique intrinsic property of simultaneous stimulation and block of secretion. The total pool of AAT synthesis in Pi MZ hepatocytes is made up by 50% of M protein and 50% of Z protein. Under conditions of clinical stimulation, both fractions undergo an increased synthesis. The M fraction is regularly exported and its serum level increases many folds. The Z fraction is retained within liver cells and gives rise to an increased amount of storage. The resulting peculiar immunostaining and EM patterns have been considered as expression of simultaneous recruitment of liver cells for synthesis and block of secretion of the mutant protein. For that reason, when originally described, the phenomenon has been called “Recruitment-Secretory Block” and has been considered sufficiently satisfactorily to explain the different morphological and serological behavior of Pi ZZ and Pi MZ individuals in response to acute phase stimuli.

The discovery of the intriguing phenomenon of M AAT and Z AAT intrahepatic heteropolymer formation, has urged us to review, retrospectively, the morphology of the study material that had led to the description of the “R-SB” phenomenon.

By running from the emerging molecular findings backwards to a critical retrospective evaluation of the published material, we found an indirect evidence that M and Z polymers can also form in vivo. Indeed, the monoclonal Z AAT antibody in Pi MZ individuals appears to stain less amount of aggregated AAT than the polyclonal antibody. The latter depicts all variants of the protein including the M. In the light of the unequivocal demonstration of the co-localization of Z and M AAT in heteropolymers [28], it is reasonable to assume that the exuberant AAT material revealed by the polyclonal antibody as compared to the monoclonal, could correspond to M AAT.

As expected, under the “R-SB” phenomenon, the M AAT as part of the heteropolymers, becomes detectable due to the increase in its intracellular concentration.

Secretion of Z polymers in cell models of disease [30] and circulating polymers have been demonstrated in AATD [31,32,44]. Mixed Z and M AAT co-expression have been found in cellular models of AATD (30) and, finally, in hepatocytes extracted from explanted Pi MZ livers [28].

Given the potential relevance of our observations, in view also of the in vitro study results, future studies should be carried out in liver tissue specimens to confirm the validity of our interpretation that quantitative evaluation could reflect qualitative meanings.

About 85% of the Z protein is retained and only some 15% is exported in circulation. Laffranchi et al. have calculated that in Pi MZ livers, M AAT comprises around 6% of the polymers subunits. That raises an important question about the percentage of M subunits rescued under the “R-SB” phenomenon. Considering that the “R-SB” phenomenon induces an increasing synthesis of both M and Z proteins, one may predict an increasing amount of M-Z polymers both within the hepatocytes and in circulation.

HHHS is an extremely rare new disease, therefore very little is known about its natural history. We know that less than 10% of fibrinogen is exported and that, in contrast with AATD, the mutant gamma chains appear to be completely retained within the cell and never found in circulation. Few available data suggest that HHHS heterozygosity behaves in a completely different way than the heterozygous AATD, but no studies have been performed so far to explore how the mutant fibrinogens respond to acute phase stimuli.

With regard to the ongoing research effort on the treatment of ERSD, a further relevant observation in our studies refers to the ultrastructural appearance of the ZAAT. Since the first ultrastructural observation by Sharp et al., the ZAAT has been always described as amorphous fluffy material within the RER [53]. In addition, we have observed also a gradual transition from the amorphous fluffy towards a denser, compact, somewhat mummified and even stone-like appearance [17]. The potentially biological meaning of the two forms has been recently discussed [54]. In the present study, mainly focused on the “R-SB” phenomenon, we have reported, especially in Pi MZ under conditions of clinical stimulation, an additional ultrastructural aspect of the retained Z protein, consisting in microfibrils or tangled structures. This feature had been reported by Lomas et al. in 1992 in an experimental work [22] in Pi ZZ material. Quite recently, Laffranchi et al. have reported hepatocytic inclusions, isolated from the liver of Pi ZZ or Pi MZ patients undergoing liver transplantation for Z AAT cirrhosis, made up by myriad tangles of filaments when observed at very high EM magnification [22,28].

These EM features are in keeping with the loop sheet mechanism of polymerization [22]. Polyacrylamide gel electrophoresis has confirmed that Z polymers, like liver inclusions in other AATD mutations with hepatic storage, are formed by non-covalent bonding and are dissociable to monomers under appropriate conditions [55,56].

The corpuscular forms of Z AAT, mainly observed in the course of the “R-SB” phenomenon in Pi MZ livers, suggest that the formation and aggregation of Z polymers are a very early event during the elongation of the polypeptide chain and their discharge into the lumen of the RER.

The same holds true for mutant fibrinogens that start aggregating during translation. The amplified acrobatic organoid fashion of fibrinogen probably reflects the complexity of the stepwise interaction and integration of the various monomers and dimers of the three chains.

The finger print and fiber glass appearance of the aggregated fibrinogen under the EM are reminding of the clot formation. Fibrinogen is converted into insoluble fibrin through a stepwise process including the assembly of single fibrin monomers into protofibrils that, in turn, aggregate into fibrin, thus yielding the stable fibrin meshwork. In the intermediate stage of the process, the fibrils display a similar metameric array as the aggregated intracellular fibrinogen.

In other words, normal secretory proteins that are soluble in the ER milieu and transparent under the EM, due to mutations, become visible under the EM after acquiring an abnormal conformation that renders them insoluble.

The Recruitment-Secretory Block phenomenon occurring in ERSD has contributed to visualize the early stage of the aggregation process of both Z AAT and abnormal fibrinogen gamma chains. The peculiar EM appearance of the aggregated proteins represent a snapshot of the crazy paving that, together with the 3-D modeling [35] could address future research towards interventions capable of simultaneously increasing the plasma levels and decreasing the hepatic storage in AATD and HHHS [57,58,59].

## Figures and Tables

**Table 1:** AAT serum levels, Pi phenotypes and immunostaining pattern in liver specimen.

Groups	N *	AAT Levels	Type II Positivity °	Polyclonal vs. Monoclonal Staining Intensity
MZ with malignancy	14	Normal/high	++++	Poly > Mono
MZ with liver disease	13	Normal (10/13)	++	Poly > Mono
MM with malignancy	30	High	No staining	No staining
ZZ with liver disease	8	Low	+/−	Poly = Mono

*: number; °“R-SB” phenomenon; >: higher than; ++++ highest degree of positivity.

## Data Availability

The data are available in the Pathology Departments of the following Hospitals: Catholic University of Leuven (Leuven), Spedali Civili of Brescia (Brescia), Istituto Giannina Gaslini, IRCCS (Genova), and Bambino Gesù Children’s Hospital, IRCCS (Rome).

## References

[1] Donohue T.M., Jennett R.B., Tuma D.J., Sorrell M.F., Zakim D., Boyer T.D. (1990). Synthesis and secretion of plasma proteins by the Liver. Hepatology—A Text Book of Liver Diseases.

[2] Shafritz D.A., Yap S.H., Strair R.K. (1979). Regulation of albumin synthesis in rat liver. Mol. Biol. Rep..

[3] Steer C.J., Ashwell G. (1986). Hepatic membrane receptors for glycoproteins. Prog. Liver Dis..

[4] Jamieson J.C., Ashton F.E. (1973). Studies on acute phase proteins of rat serum. IV. Pathway of secretion of albumin and alpha1-acid glycoprotein from Liver. Can. J. Biochem..

[5] Jamieson J.C., Ashton F.E. (1973). Studies on acute phase proteins of rat serum. 3. Site of synthesis of albumin and alpha 1-acid glycoprotein and the contents of these proteins in liver microsome fractions from rats suffering from induced inflammation. Can. J. Biochem..

[6] Parent J.B., Bauer H.C., Olden K. (1985). Three secretory rates in human hepatoma cells. Biochim. Biophys. Acta..

[7] Kushner I., Feldmann G. (1978). Control of the acute phase response. Demonstration of C-reactive protein synthesis and secretion by hepatocytes during acute inflammation in the rabbit. J. Exp. Med..

[8] Courtoy P.J., Lombart C., Feldmann G., Moguilevsky N., Rogier E. (1981). Synchronous increase of four acute phase proteins synthesized by the same hepatocytes during the inflammatory reaction: A combined biochemical and morphologic kinetics study in the rat. Lab. Investig..

[9] Callea F., Brisigotti M., Fabbretti G., Bonino F., Desmet V.J. (1992). Hepatic endoplasmic reticulum storage diseases. Liver.

[10] Palade G. (1975). Intracellular aspects of the process of protein synthesis. Science.

[11] Sabatini D.D., Kreibich G., Morimoto T., Adesnik M. (1982). Mechanisms for the incorporation of proteins in membranes and organelles. J. Cell Biol..

[12] Blobel G. (1982). Regulation of intracellular protein traffic. Cold Spring Harb. Symp. Quant. Biol..

[13] Callea F., Desmet V. (2021). The Discovery of Endoplasmic Reticulum Storage Disease. The Connection between an H&E Slide and the Brain. Int. J. Mol. Sci..

[14] Bruguera M., Bertran A., Bombi J.A., Rodes J. (1977). Giant mitochondria in hepatocytes: A diagnostic hint for alcoholic liver disease. Gastroenterology.

[15] Feldmann G., Penaud-Laurencin J., Crassous J., Benhamou J.P. (1972). Albumin synthesis by human liver cells: Its morphological demonstration. Gastroenterology.

[16] Callea F., Fevery J., Massi G., Lievens C., de Groote J., Desmet V.J. (1984). Alpha-1-antitrypsin (AAT) and its stimulation in the liver of PiMZ phenotype individuals. A “recruitment-secretory block” (“R-SB”) phenomenon. Liver.

[17] Callea F. (1983). Immunohistochemical Study on Alpha-1-Antitrypsyn. Ph.D. Thesis.

[18] Callea F., Fevery J., De Groote J., Desmet V.J. (1986). Detection of Pi Z phenotype individuals by alpha-1-antitrypsin (AAT) immunohistochemistry in paraffin-embedded liver tissue specimens. J. Hepatol..

[19] Callea F., Brisigotti M., Faa G., Lucini L., Eriksson S. (1991). Identification of PiZ gene products in liver tissue by a monoclonal antibody specific for the Z mutant of alpha 1-antitrypsin. J. Hepatol..

[20] Callea F., Massi G., De Wolf-Peeters C., Lievens C., Desmet V.J. (1982). Alpha-1-antitrypsin phenotypes in malignant lymphoma. J. Clin. Pathol..

[21] Eriksson S.G., Carlson J.A., Lindmark B.E. (1988). Serine proteinase inhibitors as acute phase reactants in liver disease. Tokai J. Exp. Clin. Med..

[22] Lomas D.A., Evans D.L., Finch J.T., Carrell R.W. (1992). The mechanism of Z alpha 1-antitrypsin accumulation in the liver. Nature.

[23] Ma M.H., Biempica L. (1971). The normal human liver cell. Cytochemical and ultrastructural studies. Am. J. Pathol..

[24] Jungermann K. (1988). Metabolic zonation of liver parenchyma. Semin Liver Dis..

[25] Zajicek G., Oren R., Weinreb M. (1985). The streaming liver. Liver.

[26] Schaffner F., Popper H. (1969). Cholestasis is the result of hypoactive hypertrophic smooth endoplasmic reticulum in the hepatocyte. Lancet.

[27] Popper H., Schaffner F. (1970). Pathophysiology of cholestasis. Hum. Pathol..

[28] Laffranchi M., Elliston E.L., Miranda E., Perez J., Ronzoni R., Jagger A.M., Heyer-Chauhan N., Brantly M.L., Fra A., Lomas D.A. (2020). Intrahepatic heteropolymerization of M and Z alpha-1-antitrypsin. JCI Insight..

[29] Janciauskiene S., Dominaitiene R., Sternby N.H., Piitulainen E., Eriksson S. (2002). Detection of circulating and endothelial cell polymers of Z and wild type alpha 1-antitrypsin by a monoclonal antibody. J. Biol. Chem..

[30] Fra A., Cosmi F., Ordoñez A., Berardelli R., Perez J., Guadagno N.A., Corda L., Marciniak S.J., Lomas D.A., Miranda E. (2016). Polymers of Z α1-antitrypsin are secreted in cell models of disease. Eur. Respir. J..

[31] Tan L., Dickens J.A., Demeo D.L., Miranda E., Perez J., Rashid S.T., Day J., Ordoñez A., Marciniak S.J., Haq I. (2014). Circulating polymers in α1-antitrypsin deficiency. Eur. Respir. J..

[32] Miranda E., Pérez J., Ekeowa U.I., Hadzic N., Kalsheker N., Gooptu B., Portmann B., Belorgey D., Hill M., Chambers S. (2010). A novel monoclonal antibody to characterize pathogenic polymers in liver disease associated with alpha1-antitrypsin deficiency. Hepatology.

[33] Callea F., Giovannoni I., Sari S., Aksu A.U., Esendagly G., Dalgic B., Boldrini R., Akyol G., Francalanci P., Bellacchio E. (2017). A novel fibrinogen gamma chain mutation (c.1096C>G; p.His340Asp), fibrinogen Ankara, causing hypofibrinogenaemia and hepatic storage. Pathology.

[34] Burcu G., Bellacchio E., Sag E., Cebi A.H., Saygin I., Bahadir A., Yilmaz G., Corbeddu M., Cakir M., Callea F. (2020). Structural Characteristics in the γ Chain Variants Associated with Fibrinogen Storage Disease Suggest the Underlying Pathogenic Mechanism. Int. J. Mol. Sci..

[35] Bellacchio E. (2020). Mutations Causing Mild or No Structural Damage in Interfaces of Multimerization of the Fibrinogen γ-Module More Likely Confer Negative Dominant Behaviors. Int. J. Mol. Sci..

[36] Lee M.J., Venick R., Bhuta S., Li X., Wang H.L. (2015). Hepatic Fibrinogen Storage Disease in a Patient with Hypofibrinogenemia: Report of a Case with a Missense Mutation of the FGA Gene. Semin Liver Dis..

[37] Callea F., Giovannoni I., Sari S., Guldal E., Dalgic B., Akyol G., Sogo T., Al-Hussaini A., Maggiore G., Bartuli A. (2017). Fibrinogen Gamma Chain Mutations Provoke Fibrinogen and Apolipoprotein B Plasma Deficiency and Liver Storage. Int. J. Mol. Sci..

[38] Brennan S.O., Wyatt J., Medicina D., Callea F., George P.M. (2000). Fibrinogen brescia: Hepatic endoplasmic reticulum storage and hypofibrinogenemia because of a gamma284 Gly-->Arg mutation. Am. J. Pathol..

[39] Brennan S.O., Maghzal G., Shneider B.L., Gordon R., Magid M.S., George P.M. (2002). Novel fibrinogen gamma375 Arg-->Trp mutation (fibrinogen aguadilla) causes hepatic endoplasmic reticulum storage and hypofibrinogenemia. Hepatology.

[40] Medicina D., Fabbretti G., Brennan S.O., George P.M., Kudryk B., Callea F. (2001). Genetic and immunological characterization of fibrinogen inclusion bodies in patients with hepatic fibrinogen storage and liver disease. Ann. N. Y. Acad. Sci..

[41] Redman C.M., Xia H. (2001). Fibrinogen biosynthesis. Assembly, intracellular degradation, and association with lipid synthesis and secretion. Ann. N. Y. Acad. Sci..

[42] Janero D.R., Siuta-Mangano P., Miller K.W., Lane M.D. (1984). Synthesis, processing, and secretion of hepatic very low density lipoprotein. J. Cell Biochem..

[43] Stein O., Sanger L., Stein Y. (1974). Colchicine-induced inhibition of lipoprotein and protein secretion into the serum and lack of interference with secretion of biliary phospholipids and cholesterol by rat liver in vivo. J. Cell Biol..

[44] Laffranchi M., Berardelli R., Ronzoni R., Lomas D.A. (2018). Heteropolymerization of alpha-1-antitrypsin mutants in cell models mimicking heterozygotes. Hum. Mole Genet..

[45] Ronzoni R., Heyer-Chauhan N., Fra A., Pearce A.C., Rüdiger M., Miranda E., Irving J.A., Lomas D.A. (2021). The molecular species responsible for α1 -antitrypsin deficiency are suppressed by a small molecule chaperone. FEBS J..

[46] Kruse K., Dear A., Kartenbrund E.R., Crum B.E., George P.M., Brennan O.S. (2006). Mutant fibrinogen clearance of Endoplasmic Reticulum-Associated Protein Degradation and autophagy: An explanation for liver disease. Am. J. Pathol..

[47] Maggiore G., Nastasio S., Sciveres M. (2011). Long-term outcome of liver disease-related fibrinogen aguadilla storage disease in a child. J. Pediatr. Gastroenterol. Nutr..

[48] Carlson J.A., Rogers B.B., Sifers R.N., Finegold M.J., Clift S.M., De Mayo F.J., Bullock D.W., Woo S.L. (1989). Accumulation of PiZ alpha 1-antitrypsin causes liver damage in transgenic mice. J. Clin. Investig..

[49] Rudnick D.A., Liao Y., An J.K., Muglia L.J., Perlmutter D.H., Teckman J.H. (2004). Analyses of hepatocellular proliferation in a mouse model of alpha-1-antitrypsin deficiency. Hepatology.

[50] Giovannoni I., Callea F., Stefanelli M., Mariani R., Santorelli F.M., Francalanci P. (2015). Alpha-1-antitrypsin deficiency: From genoma to liver disease. PiZ mouse as model for the development of liver pathology in human. Liver Int..

[51] Francalanci P., Santorelli F.M., Saccani S., Bonetti M.F., Medicina D., Coni P., Faa G., Callea F. (2009). Z and Mmalton-1-antitrypsin deficiency-associated hepatocellular carcinoma: A genetic study. Liver Int..

[52] Callea F. (1988). Natural history of hepatocellular carcinoma as viewed by the pathologist. Appl. Pathol..

[53] Sharp H.L., Bridges R.A., Krivit W., Freier E.F. (1969). Cirrhosis associated with alpha-1-antitrypsin deficiency: A previously unrecognized inherited disorder. J. Lab. Clin. Med..

[54] Callea F., Fabbretti G., Bonetti M., Brisigotti M., Desmet V.J., Schmid R., Bianchi L., Gerok W., Maier K.P. (1993). Alpha-1-Antitrypsin Deficiency in Extrahepatic Manifestations in Liver Diseases.

[55] Eriksson S., Larsson C. (1975). Purification and partial characterization of pas-positive inclusion bodies from the liver in alpha 1-antitrypsin deficiency. N. Engl. J. Med..

[56] Bathurst I.C., Travis J., George P.M., Carrell R.W. (1984). Structural and functional characterization of the abnormal Z alpha 1-antitrypsin isolated from human liver. FEBS Lett..

[57] Wewers M.D., Gadel J.E., Keugh R.A., Fells G.A., Cristal R.G. (1986). Evaluation of danazol for patients with Pi ZZ alpha-1-antitrypsin deficiency. Am. J. Resp. Dis.

[58] Zhang X., Pham K., Li D., Schutte R.J., Gonzalo D.H., Zhang P., Oshins R., Tan W., Brantly M., Liu C. (2019). A Novel Small Molecule Inhibits Intrahepatocellular Accumulation of Z-Variant Alpha 1-Antitrypsin in Vitro and in Vivo. Cells.

[59] Lomas D.A., Irving J.A., Arico-Muendel C., Belyanskaya S., Brewster A., Brown M., Chung C.W., Dave H., Denis A., Dodic N. (2021). Development of a small molecule that corrects misfolding and increases secretion of Z α1 -antitrypsin. EMBO Mol. Med..

